# Intersection of pathological tau and microglia at the synapse

**DOI:** 10.1186/s40478-019-0754-y

**Published:** 2019-07-05

**Authors:** Thomas Vogels, Adriana-Natalia Murgoci, Tomáš Hromádka

**Affiliations:** 1 Axon Neuroscience R&D Services SE, Bratislava, Slovak Republic; 2 Institute of Neuroimmunology, Slovak Academy of Sciences, Bratislava, Slovak Republic

**Keywords:** Tau pathology, Tau immunotherapy, Microglia, Astrocytes, Synaptic dysfunction, Complement, Neurodegeneration, Neuroinflammation, APOE4, TREM2

## Abstract

Tauopathies are a heterogenous class of diseases characterized by cellular accumulation of aggregated tau and include diseases such as Alzheimer’s disease (AD), progressive supranuclear palsy and chronic traumatic encephalopathy. Tau pathology is strongly linked to neurodegeneration and clinical symptoms in tauopathy patients. Furthermore, synapse loss is an early pathological event in tauopathies and is the strongest correlate of cognitive decline. Tau pathology is additionally associated with chronic neuroinflammatory processes, such as reactive microglia, astrocytes, and increased levels of pro-inflammatory molecules (e.g. complement proteins, cytokines). Recent studies show that as the principal immune cells of the brain, microglia play a particularly important role in the initiation and progression of tau pathology and associated neurodegeneration. Furthermore, AD risk genes such as Triggering receptor expressed on myeloid cells 2 (*TREM2*) and Apolipoprotein E (*APOE*) are enriched in the innate immune system and modulate the neuroinflammatory response of microglia to tau pathology. Microglia can play an active role in synaptic dysfunction by abnormally phagocytosing synaptic compartments of neurons with tau pathology. Furthermore, microglia are involved in synaptic spreading of tau – a process which is thought to underlie the progressive nature of tau pathology propagation through the brain. Spreading of pathological tau is also the predominant target for tau-based immunotherapy. Active tau vaccines, therapeutic tau antibodies and other approaches targeting the immune system are actively explored as treatment options for AD and other tauopathies. This review describes the role of microglia in the pathobiology of tauopathies and the mechanism of action of potential therapeutics targeting the immune system in tauopathies.

## Introduction

### The role of microglia in tauopathies

Pathological tau protein is observed a wide range of neurodegenerative disorders (NDD) and is the key defining feature of a heterogeneous class of diseases called tauopathies. Alzheimer’s disease (AD) is the most common tauopathy - affecting approximately 45 million people worldwide – and is additionally characterized by extracellular plaques composed of amyloid beta (Aβ) [265]. Less common tauopathies include Picks’ disease (PiD), corticobasal degeneration (CBD), progressive supranuclear palsy (PSP), argyrophilic grain disease (AGD), and chronic traumatic encephalopathy (CTE). In AD and other tauopathies, tau pathology closely correlates with neurodegeneration and functional decline [11, 115, 147, 211, 232]. Additionally, tauopathies are characterized by early synaptic dysfunction. Tau-induced damage in synaptic compartments ultimately leads to major synapse loss, which is the closest correlate of cognitive decline [76, 148, 263, 264]. Furthermore, synaptic connections are the principal sites at which pathological tau can spread from diseased to healthy neurons – a process which is thought to underlie the progressive nature of tau pathology throughout the brain [218]. Tauopathies are also characterized by reactive gliosis and an increase in inflammatory molecules such complement proteins and pro-inflammatory cytokines – collectively referred to as neuroinflammation [65, 79, 106, 144, 205, 233, 244, 267, 271, 272, 274]. The purpose of the neuroinflammatory state is to remove the cause (e.g. pathogens, protein aggregates, damaged cells) and return the tissue to homeostasis. However, it is not clear if neuroinflammation in tauopathies is mostly protective or damaging and how this depends on disease stage.

Multiple cell types can have immune functions in the brain, for example microglia, astrocytes, perivascular macrophages, meningeal macrophages, choroid plexus macrophages, and infiltrating peripheral myeloid cell types [304]. However, microglia are of particular interest as they are the principal macrophages of the CNS and exciting recent research has shown novel roles for these immune cells in both health and disease. Additionally, genome-wide association studies (GWAS) have identified several late onset AD (LOAD) risk variants that are found in proteins that are predominantly expressed in the innate immune system and microglia (e.g. APOE, TREM2, ABCA7, CD33, CR1) [207]. This strongly implicates microglia as central players in the development of LOAD [124]. Given the central role of tau pathology in AD and other tauopathies, there is now increasing interest in how microglia are involved in the pathobiology of tau protein. It is currently unclear if altered microglial function is a cause, consequence, or contributor to tau pathology. Secreted factors from microglia may lead to initiation of tau aggregation in neurons [116]. Microglia may be also involved in tau-induced synapse loss and tau spreading, and play an important role in the mechanism of action of tau immunotherapy and other therapeutics aimed at treating tauopathies [12, 99, 135, 192]. This review provides an overview of how interaction of tau pathology and microglia leads to synaptic dysfunction in tauopathies. Furthermore, we provide an overview of the published preclinical in vivo studies of tau immunotherapy and immune-related pathways for the treatment of tauopathies.

### Tau pathology

Tau is an abundant protein that is predominantly expressed in the axonal compartment of neurons, but also at lower levels in oligodendrocytes and astrocytes [16]. The main function of tau is to regulate the assembly, nucleation and bundling of microtubules and to modulate axonal transport [122]. In addition, recent research suggests that tau may also have a multitude of other physiological functions [282]. Tau protein is encoded by the microtubule-associated protein tau *(MAPT*) gene on chromosome 17q21.31, and this gene can be mutated, inverted, duplicated, and abnormally methylated. All these modifications have been associated with increased risk of developing tauopathy and the genetic evidence therefore clearly links tau to neurodegeneration [18, 138, 139, 176].

**Fig. 1:**
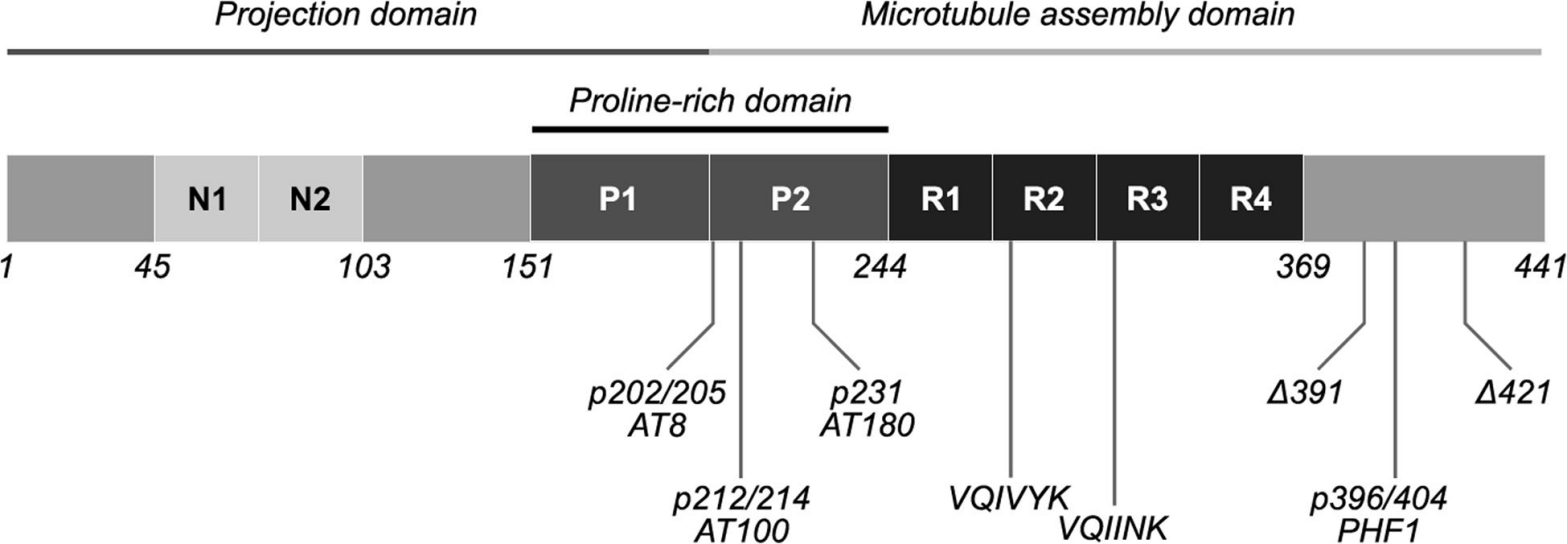
Major tau domains and phosphorylation sites. The amino acid sequence of the longest isoform of tau protein (2N4R, 1–441aa) in the central nervous system can be roughly divided into the projection domain on the N-terminal and the microtubule assembly domain on the C-terminal half of the protein. Tau can have up to two inserts in the N-terminal (here shown as N1, N2) and three or four repeats on the C-terminal (R1, R2, R3, R4). These combinations lead to a total of six different isoforms in the central nervous system. The VQIVYK sequence in R2 and VQIINK sequence in R3 are important for aggregation of tau. Several important phosphorylation sites that are associated with tau pathology are shown (p202/205, p212/214, p231, p396/404). These sites are targets for widely used antibodies such as AT8 or PHF1. Several C-terminal truncations have been identified that promote aggregation. Two wellcharacterized truncations are shown here (Δ391 and Δ421)

The human brain contains 6 isoforms generated by alternative splicing of exons 2, 3 and 10 of the *MAPT* gene [314]. Tau can have either 0, 1 or 2 N-terminal inserts and either 3 or 4 pseudo-repeats (R), resulting in isoforms ranging from 352 to 441 amino acids (aa) (36.7–45.9 kDa) (Fig. 1) [112]. Tau protein can be subdivided into several domains: a structurally disordered N-terminal, the proline rich mid-domain and a highly conserved C-terminal which includes microtubule binding repeats (MTBR). Tau is also subject to a wide range of post-translational modifications (PTMs) (e.g. phosphorylation, acetylation, truncation), which alter its structure, function, and subcellular localization [171, 325]**.** The six isoforms in combination with the multitude of potential PTMs make the biology of tau extraordinarily complex. Belonging to the class of natively unfolded or intrinsically disordered proteins, tau proteins lack clearly defined secondary and tertiary structures.

The MTBR of tau contains two hexapeptides that can form intermolecular beta sheet rich structures: aa275–280 (VCIINK) in R2 and aa306–311 (VQIVYK) in R3 [307, 308]. Pathological conformations of tau can interact with physiological tau, leading to aggregation and ultimately formation of highly structured insoluble fibrils which deposit into the cell as neurofibrillary tangles (NFTs). This process is referred to as templated misfolding, seeded nucleation, or simply seeding [97]. As tau is a highly soluble protein and the initial aggregation phase is thermodynamically unfavorable, it is currently unclear how tau shifts from its dynamic physiological structure to a misfolded monomer that is prone to aggregation [212, 270]. Specific patterns of PTMs may change the conformation of the protein, causing tau to become seed-competent [62, 77]. Moreover, dynamic phosphorylation of the residues in the MTBR or flanking regions regulates the affinity of tau for tubulin and hyperphosphorylation may thereby increase the pool of free tau available for aggregation [160]. The phosphorylation of tau is regulated by both kinases (e.g. cdk5, GSK-3β, p38-MAPK) and phosphatases (e.g. PP2A) [142]. Phosphorylation at a number of sites on tau has been linked to tau pathology (e.g. Ser202/Thr205, Thr212/Ser214, Thr231, Ser396/Ser404, Fig. 1) [314]. Abnormal cleavage can potentially play an important role in tauopathies, as several truncated fragments have an increased propensity for aggregation and their overexpression leads to neurofibrillary pathology in rodents [87, 329]. As will be discussed later in this review, factors secreted from microglia can lead to abnormal patterns of PTMs and may therefore play a role in the initiation of tau aggregation.

Smaller tau oligomers are still soluble and can mislocalize to the somatodendritic compartment to cause toxicity throughout the cell [325]. For this reason, intracellular tau oligomers are also the most toxic species for synapses [120]. In addition to causing intracellular toxicity, tau oligomers and short fibrils can be secreted into the extracellular space and taken up by healthy neurons [98, 121, 162]. This process may be of critical importance as it is thought to underlie the progression of tau pathology throughout the brain. Interestingly, it has already been observed in the classical Braak staging scheme that the progression of tau pathology seems to occur along neuronal connections [43]. It has been demonstrated using a variety in vitro and in vivo approaches that tau pathology predominantly spreads along synaptic connections [48, 73, 318]. Recent studies have made significant progress in showing that this also occurs in the brain of Alzheimer’s patients: seed-competent tau is present in axons of white matter tracts and synaptosomes, and tau seeding occurs in synaptically connected areas before the occurrence of hyperphosphorylated tau in these regions [78, 100, 158, 159]. It is currently unclear what the major mechanism of synaptic tau secretion is, but the evidence so far suggests: (1) release from synaptic vesicles [242] (2) secretion in extracellular vesicles such as exosomes [241, 256, 313] and ectosomes [80], (3) direct translocation across the membrane [157, 208] or (4) tunneling nanotubes [1, 295]. Similarly, several tau uptake mechanisms have been identified which are not mutually exclusive: (1) bulk endocytosis [98, 121, 259, 317] macropinocytosis by heparin sulfate proteoglycans [84, 132, 162, 248, 288, 328] or (3) clathrin-mediated endocytosis [49, 82]. After tau seeds enter the neuron they can seed physiological monomers, thereby propagating the disease process [85].

Neuronal stress or neuronal damage induced by intracellular tau pathology can also impact nearby immune cells, such as microglia [174]. Furthermore, microglia can be affected by extracellular tau secreted by neurons with tau pathology and tau filaments leaking from dying cells [257]. Microglia may also be directly involved in tau-induced synapse loss and synaptic spreading of tau pathology [12, 75]. Understanding how microglia contribute to synaptic dysfunction is therefore of critical importance and will be discussed in more detail below.

### Microglia and their role at the synapse

Microglia are the tissue resident macrophages in the brain and originate from yolk-sac-derived erythro-myeloid progenitors [113]. Their unique identity - which distinguishes them from other macrophages in the brain - is the result of this ontogeny and the characteristic micro-environment in the brain [25]. Once microglia have established themselves in the brain during development, their colony is maintained through continuous self-renewal [3, 137]. Microglia constantly scan the extracellular environment for signs of damage or infection and rapidly direct their processes to local brain injury [70, 223]. Microglia respond to so-called damage and pathogen associated molecular patterns via a variety of surface receptors [129]. Additionally, microglial filipodia make contact with neurons, astrocytes, and perivascular cells [303]. Neurons secrete a multitude of signaling molecules that influence the behavior of microglia [32]. Microglia can for example respond to both inhibitory and excitatory neurotransmitters and their processes interact with neuronal synapses in an activity-dependent manner [83, 94, 298]. This process may have functional consequences as microglia have been shown to be involved in activity-dependent formation and removal of synapses [316].

During neurodevelopment, microglial contact induces synapse formation in the cortex [213]. Furthermore, developmental pruning by microglial phagocytosis is critical for normal brain development [234, 262]. Knockout of the chemokine (C-X3-C motif) ligand 1 (Cx3cl1) receptor leads to reduced microglial synaptic pruning, altered synaptic function, neural connectivity, and social behavior [39, 136, 234, 326]. It is unclear how loss of CX3C chemokine receptor 1 (Cx3cr1) leads to pruning deficits, but it is possible that the chemokine Cx3cl1 acts as a soluble “find-me” signal for microglia. In addition, P2Y12 purinergic receptors may also act as receptors that respond to “find me” signals from synapses. P2Y12 receptors are required for process outgrowth to damaged tissue [125, 193] and also modulate synaptic plasticity in visual cortex [280]. A more direct pathway is a surprising new role for the complement system. Complement initiation factor C1q tags synapses for removal in an activity dependent manner. This subsequently leads to deposition of complement component 3 (C3) and microglial phagocytosis via complement receptor 3 (C3R) [262]. This pathway seems to be reactivated under neurodegenerative conditions and this will be discussed in later sections of this review. A comprehensive understanding of the signals that lead to localization of C1q at synapses is still missing, but it is known that microglia are the dominant source of C1q [92]. Additionally, astrocytic TGF-β signaling can induce C1q expression in developing retinal neurons and blocking this pathways blocks synapse removal [31]. Astrocytes also secrete interleukin-33, which acts on microglial interleukin 1 receptor-like 1 to promote synapse phagocytosis [302]. Microglial synapse phagocytosis via triggering receptor expressed on myeloid cells 2 (TREM2) – which is encoded by a LOAD risk gene - also plays a role in normal development of neural circuits [88]. Developmental synaptic pruning by microglia is a tightly regulated process as microglia also respond to “don‘t eat me” signals such as cluster of differentiation 47 (CD47) to prevent excess pruning [180].

Microglia also play an important role in maintaining synaptic structure and function later in life. Microglia are for example required for maintenance of synaptic structure and synaptic transmission in the adult retina [312]. Microglia-synapse contacts were also shown to enhance synaptic activity and promote neuronal network synchronization [4]. Furthermore, activated microglia can protect the adult brain by migrating towards inhibitory synapses and displacing them from cortical neurons [55]. Interestingly, microglia play a role in the adult brain by learning dependent synapse formation via secretion of brain-derived neurotrophic factor (BDNF) [235]. Microglial cytokines interleukin (IL)-1 beta (1B), IL-2, IL-6, IL-8, IL18, interferon (IFN)-alpha, INF-gamma and tumor necrosis factor alpha (TNF-a) are all involved in synaptic plasticity, learning, and memory [225]. Low levels of even pro-inflammatory cytokines might therefore be necessary for normal synaptic function. Microglia thus have important physiological functions at the synapse in both the developing and adult brain.

### Microglia in the aging brain

When trying to understand the effects of pathological protein aggregates such as tau pathology on the brain, it is important to note that in humans these effects are often superimposed on the normal effects of aging. In rats, for example, viral delivery of tau protein to young and aged animals led to more microgliosis, neuronal loss, and behavioral deficits in the aged group [166]. It is therefore also important to understand the normal alterations of microglia in the aging brain. For example, a somatic mutation in microglia precursor cells leads to late-onset neurodegeneration [201], which suggests that genetic phenotypes of microglia can manifest themselves in the context of the aging. It is therefore possible that the effects of late onset AD risk mutations in proteins expressed in microglia only become apparent at advanced age. Indeed, haploinsufficiency of AD risk gene *TREM2* only leads to impaired response of microglia to injury in old mice [261]. Furthermore, in old age, microglia operate in an aged environment. For example, age-related myelin fragmentation overloads the microglial lysosomal system and contributes to microglial senescence and immune dysfunction in aging [255].

Microglia not only respond to the aging cells around them but also display signs of senescence themselves in the aging human brain [231, 292]. In vivo imaging of young, adult, and very old mice shows changes in morphology and behavior in addition to a slight increase in cell density [126]. The transcriptional microglial phenotype in aging and chronic neurodegeneration is different from acute microglial activation by lipopolysaccharide (LPS) [133]. Microglial genes that encode proteins involved in the scanning of the brain parenchyma - the so-called ‘sensome’ – change their expression in aging [130]. Although mouse and human microglia have a large overlap in expression patterns, these genetic networks start to diverge in aging [101, 117]. The genes that are different in aging are associated with actin dynamics and the sensome, indicating that mouse and human microglia age quite differently. Furthermore, microglia also show regional variation in gene expression in aging, indicating that some brain regions may be more vulnerable to aging of the innate immune system [118]. Taken together, microglial senescence may impair their ability to keep the aging brain clean.

## Bidirectional effects of tau pathology and microglial neuroinflammation

### The effects of tau pathology on microglia

**Fig. 2:**
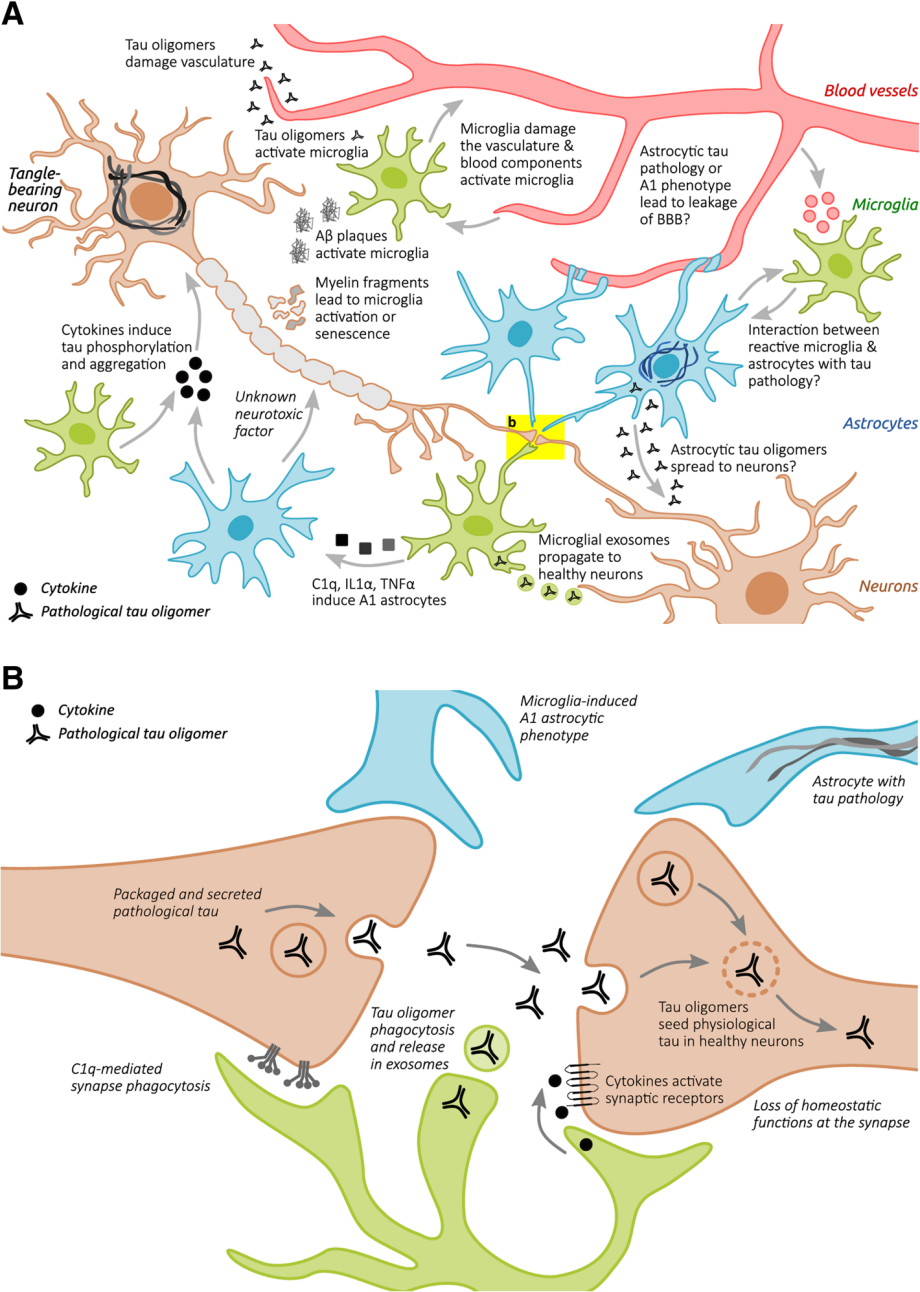
**a** Several cell types are involved in tau-induced neuroinflammation. Neurons with tau pathology exposing phosphatidylserines can be live phagocytosed by microglia. Neuronal tau pathology also induces neuroinflammation by shedding myelin fragments, secreting stress factors, tau oligomers, or via other unknown pathways. In Alzheimer’s disease – the most common tauopathy – extracellular amyloid plaques also induce neuroinflammation. Tau oligomers can damage the vasculature directly, or indirectly via microglia-induced neuroinflammation or alterations of astrocytic functions at the vasculature. All these events can potentially lead to exacerbation of the neuroinflammatory state, which in turn can aggravate tau pathology via proinflammatory cytokines. Microglia can also induce a neurotoxic “A1” phenotype in astrocytes which directly leads to neurodegeneration. Astrocytes in primary tauopathies can also accumulate tau, which can lead to mild changes in the vasculature and possibly impact microglia and synaptic function. **b** Microglia and astrocytes play an important role in tau-induced synaptic dysfunction. Microglia can phagocytose synapses from neurons with tau pathology via the classical complement pathway. Microglia can also phagocytose secreted tau oligomers and spread them to healthy neuron in exosomes. Microglia in the healthy brain also play an important role in synapse homeostasis, for example via the secretion of cytokines or secretion of growth factors. Tau pathology could alter these homeostatic functions and lead to possible toxic gain-of-function. Astrocytes also play a critical role in synaptic function, for example by taking up extracellular glutamate, release of gliotransmitters that act on synaptic receptors, and secretion of factors that promote synapse assembly. Microglia in tauopathies can also alter the homeostatic functions of astrocytes, possibly leading to synaptic toxicity. Astrocytes with tau pathology can potentially also have deleterious effects on synaptic functions, but this is not yet studied and the role of microglia is therefore unclear

In AD, microglia were previously predominantly studied in the context of plaque pathology and plaque-associated microglia were indeed already observed by Alois Alzheimer [7]. However, reactive microglia, reactive astrocytes, and inflammation-associated molecules are also observed around neurofibrillary tangles (NFTs) and ghost NFTs in AD brains [65, 79, 119, 233, 244, 269, 271, 274]. Furthermore, the same is also observed in primary tauopathies such as PiD, CBD, PSP, Guam Parkinson, Anti-IgLON5 disease [21, 56, 105, 106, 127, 128, 144, 237, 267], and tau transgenic animals [13, 140, 260, 289, 322, 331, 332]**.** As will be described in more detail later, tau pathology is also robustly associated with activation of classical complement cascade and the release of pro-inflammatory cytokines such as IL1B, IL6 and TNFa [182]. A variety of factors can potentially mediate tau-induced neuroinflammation (Fig. 2a).

The most obvious one is that tau aggregates directly activate microglia. Tau oligomers co-localize with microglia, astrocytes, and pro-inflammatory cytokines in the brains of tauopathy patients and transgenic mice [222]. When applied in vitro, tau monomers, oligomers, and fibrils directly cause alterations in microglial morphology and secretion of pro-inflammatory cytokines [216, 238]. Microglia have the capacity to phagocytose tau aggregates in vitro and in vivo [12, 44, 73, 74, 99, 135, 192] and recent research shows that the process is partly dependent on Cx3cr1 receptors [37, 38]. Although the phagocytic capacity of microglia for tau aggregates seems to be relatively modest [135, 196], tau aggregates are consistently found in reactive microglia in patient brains [135, 229]. It is unclear if tau aggregates cause microglial activation after phagocytosis or if they are recognized by microglial surface receptors leading to pro-inflammatory cytokine release. Overexpression of full-length tau in microglia causes their activation, but it is unclear how this finding relates to the uptake of tau by microglia in the brain [310].

Interestingly, application of AD-derived soluble tau to cultured microglia causes their degeneration [251, 257] and dystrophic microglia in the aged marmoset often contain hyperphosphorylated tau [249]. Indeed, during aging and AD, altered cytoskeleton, morphology, and senescence of microglia have stronger correlation with tau pathology than microglial activation [17, 72, 249, 291, 292, 297]. It is therefore possible that microglia first have the ability to phagocytose extracellular tau, but they are ultimately not able to keep up with degrading insoluble material around them. This leads them to become dystrophic and lose their normal homeostatic functions [135, 290]. Microglia also show regional variation in clearance of dying neurons and dysfunctional synapses, which may contribute to regional vulnerability to tauopathy [15]. Neuronal tau pathology leads to accumulation of senescent microglia and astrocytes and removal of these senescent cells from a mouse model of neuronal tauopathy led to decreased tau pathology and improved cognition [47]. The presence of dysfunctional glial cells can thus directly contribute to neuronal tau pathology.

Neurons that are trying to cope with tau pathology express factors such as Cx3Cl1 acting on microglial receptor Cx3cr1 [174]. This signaling mechanism limits overactivation of microglia, and these types of pathways are therefore referred to as immune checkpoints [129]. In aged mice or animal models with Aβ plaque deposition, receptors for immune checkpoints are downregulated in microglia [161]. Once tau-induced degeneration of the neuron progresses, intracellular components, myelin debris, and intracellular tau aggregates may activate microglia. Live neurons with tau filaments expose phosphatidylserines, which act as an “eat-me” signal to microglia. Microglia then secrete the opsonin milk-fat-globule EGF-factor-8 and nitric oxide, leading to live phagocytosis of the neuron [44]. It is currently unclear, however, if this is process is harmful or helpful. Microglial phagocytosis of stressed-but-viable neurons may lead to cognitive decline via disintegration of neuronal networks [46]. On the other hand, preventing phagocytosis of live neurons or neuronal compartments with tau filaments may cause inflammation and leakage of aggregated tau which can spread to healthy neurons [293].

The effects of tau pathology on microglia may also be mediated by the vasculature. Tau pathology leads to vascular inflammation and alterations of blood vessels [27, 151, 163, 194, 210], which may be caused by accumulation of tau oligomers and fibrils in the microvasculature [34, 52, 210]. Indeed, early AD is already associated with cerebrospinal fluid (CSF) markers of cerebrovascular inflammation which is associated with phosphorylated tau [149]. Additionally, tau-induced neuroinflammation can damage the blood brain barrier (BBB) which may in turn exacerbate inflammation as blood components activate microglia [71, 170]. Furthermore, tau pathology was reported to lead to infiltration of peripheral immune cells, via secretion of microglial chemokine CCL3 and increased expression of endothelial signaling molecules [175, 195]. This process was associated with neuroinflammation and depleting peripheral T-cells with an anti-CD3 antibody reduced tau pathology-induced expression of pro-inflammatory cytokines and cognitive deficits. Microglia may therefore adversely affect the vasculature downstream of tau pathology, but could in turn also get affected by vascular abnormalities and alterations in the BBB. Interestingly, astrocytes also contain tau inclusions in primary tauopathies such as PSP, CBD, PiD, as well as in the aging brain [131]. A mouse model of astrocytic tau pathology contains tau inclusions in the astrocytic endfeet associated with vasculature. This is accompanied by accumulation of IgG and albumin around the blood vessels, indicative of mild BBB disruption that may in turn lead to microglial activation [95].

### Microglia contribute to tau pathology

Whether inflammation is a cause, a contributor, or a consequence of tau pathology is one of the central questions relating to the role of microglia in tauopathies [330]. Several studies have used genetic approaches in mice to examine the relationship between microglia, inflammation, and tau pathology. As mentioned previously, Cx3cl1 acts on microglial receptor Cx3cr1 to limit microglia-induced neuroinflammation. Cx3cl1 overexpression in a tauopathy mouse model decreases tau hyperphosphorylation of tau, neurodegeneration, and cognitive deficits – likely by suppressing microglial activation via Cx3cr1 [89, 221]. The opposite effect was observed in CX3CR1 receptor knockouts [22, 29, 178, 199]. Curiously, knockout of tau also rescued inflammation-mediated neurodegeneration in mice lacking the Cx3cr1 receptor [198]. This indicates that endogenous tau may protect against inflammation and its downstream effects via a yet-unknown mechanism. The deletion of small GTPase *RhoA* specifically from microglia led to microglial activation, astrogliosis, increased transcription levels of pro-inflammatory cytokines, neurodegeneration, and accumulation of hyperphosphorylated tau in wild-type mice [281]. This suggests that microglia-induced inflammation could not only aggravate existing tau pathology, but potentially also initiate accumulation of hyperphosphorylated tau. In addition to the effects of specific genes, transgenic animals bred on a background that is more prone to neuroinflammation have increased neurofibrillary pathology, despite similar expression levels of truncated tau [289]. These studies show that inflammation can directly lead to initiation or aggravation of tau pathology and its associated consequences.

Administration of anti- or proinflammatory stimuli or compounds has been used to demonstrate that microglia and inflammation are linked to tau pathology. Treatment of mouse models of tauopathy with anti-inflammatory drugs led to a decrease in tau pathology [104, 322]. Furthermore, depletion of microglia with drugs that block colony-stimulating factor-1, which is critical for microglial survival, led to a decrease in accumulation of hyperphosphorylated tau in a mouse model of tauopathy [12], but not in the 3xTG mouse model that develops both tau pathology and Aβ plaques [68]. Additionally, reduction of microglia using the same approach in an aged aggressive tauopathy model did not lead to changes in tau pathology or neurodegeneration [26]. Approaches to reduce inflammation in mouse models in tauopathy will be described in more detail in later sections of this review and are summarized in Table 1.

**Table 1:** Pharmacological approaches to target microglial inflammation in mouse models of tauopathy

Publication	Target (drug name)	Potential mechanism	Mouse line, age at start of study, administration schedule	Results
Yoshiyama (2007) [322]	Calcineurin (FK506/Tacrolimus)	Immunosuppression	PS19 (1N4R/P301S) 2M, drug in drinking water until 6M or 12M	↓atrophy/neurodegeneration, ↓neuroinflammation, ↓ tau pathology, ↑survival
Noble (2009) [226]	Multiple (Minocycline)	Anti-inflammatory	hTau (6 isoforms), 3-4M or 12M, 14 days, daily i.p.	↓caspase activity, ↓truncated tau, ↓p-tau, ↓aggregated tau
Garwood (2010) [104]	Multiple (Minocycline)	Anti-inflammatory	hTau 3-4M, 14 days daily i.p.	↓astrogliosis, ↓pro-inflammatory cytokines
Laurent (2017) [175]	CD3 (145-2C11)	Depletion of T-cells	THY-Tau22 (4R1N/G272V & P301S) 4M, every 2 weeks i.p. until 9M	↓spatial memory deficits, ↓neuroinflammation, normalization of synaptic plasticity, NC tau pathology
Asai (2015) [12]	CSF1 (PLX3397)	Depletion of microglia	PS19 3.5M, WT injected with Tau AAV, drug in food for 1M	↓tau spreading (AAV), ↓p-tau (PS19), ↓pro-inflammatory cytokines, rescue of network hypoexcitability
Bennett (2018) [26]	CSF1 (PLX3397)	Depletion of microglia (partial)	Tg4510 (0N4R/P301L) 12M, drug in food for 3M	NC tau pathology, NC atrophy, NC blood vessel morphology, NC astrocyte activation
Dejanovic (2018) [75]	C1q (M1)	Inhibition complement cascade, reduction synapse phagocytosis	PS19 9M, 1x hippocampal injection	↓synapse phagocytosis, ↓synapse loss
Litvinchuk (2018) [190]	pSTAT3 (SH-4-54)	Inhibition of signalling downstream of C3aR	PS19 7M, 3x/week i.p. until 9M	↓neuroinflammation, ↓tau pathology
Bussian (2018) [47]	Bcl-2, Bcl-XL, Bcl-w (ABT263/Navitoclax)	Removal of senescent glia	PS19 weaning age, cycles of 5D daily (oral galvage) with 16D rest until 6M	↓P-tau
Giannopoulos (2015) [110]	5-lipoxygenase (Zileuton)	Reduction leukotriene-induced inflammation	hTau 3M, drug 3x per week in drinking water until 10M	↓P-tau, ↓neuroinflammation, ↓synapse loss, rescue of synaptic deficits, rescue of cognitive deficits
Giannopoulos (2018) [108]	5-lipoxygenase (Zileuton)	Reduction leukotriene-induced inflammation	PS19 3M, drug 3x per week in drinking water until 10M	↓P-tau, ↓neuroinflammation, ↓synapse loss, rescue of cognitive deficits
Stancu (2019) [284]	NLRP3 inhibitor (MCC950)	Inflammasome inhibition	PS19 (injected with PFF) 3M, i.c.v. with osmotic pumps for 7W	↓tau pathology, ↓microgliosis

Induction or exacerbation of inflammation is also likely linked to tau pathology. Both administration of LPS and virus-induced inflammation led to increased hyperphosphorylated and insoluble tau in 3xTg mice and this effect could be rescued by blocking the kinase GSK-3B [165, 294]. LPS also accelerates accumulation of hyperphosphorylated tau in the aggressive rTg4510 tauopathy model, but this was not associated with more Gallyas-positve NFTs [177]. Importantly, LPS was even shown to induce accumulation of phosphorylated tau in wild-type mice [102, 250]. In addition, administration of viral mimic polyriboinosinic-polyribocytidilic acid also led to peripheral inflammation, release of pro-inflammatory cytokines, missorting of tau to the somatodendritic compartment, and accumulation of hyperphosphorylated tau in wild-type mice [173]. Thus, neuroinflammation could not only exacerbate ongoing tau pathology but potentially also lead to the earliest pathological events of tau pathology. How microglial inflammation might worsen or even possibly initiate tau pathology is an important question, which will be the topic of the next sections.

### The role of the complement pathway in tau pathology

One consistently upregulated pathway in tauopathies is complement [305]. The complement system is part of the innate immune system and enhances the ability of antibodies and phagocytes to clear pathogens and damaged cells. Complement consists of three potential initiating pathways that all converge on the formation of a C3 convertase which cleaves C3 into C3a and C3b [305], which then cleaves C5 into C5a and C5b. C3a and C5a are anaphylatoxins that play an important role in attracting immune cells and increasing inflammation [305]. C3b on the other hand binds to pathogens or damaged cells and interacts with C3R on phagocytes such as microglia to enhance phagocytosis [305]. C5b plays an import role in the membrane-attack-complex (MAC). The MAC disrupts the integrity of the cell membrane and leads to death and lysis of the cell [305].

The complement pathway has been studied at multiple levels in the context of tau pathology. For example, overexpression of natural C3 inhibitor sCrry was found to decrease tau pathology [45]. Accordingly, knocking out C3aR - the receptor for the chemo-attractant peptide C3a (C3aR) – led to the rescue of hyperphosphorylated and misfolded tau accumulation [190]. The tauopathy mice without the C3aR also had almost no signs of neuroinflammation, synapse loss, neurodegeneration, and cognitive deficits [190]. More downstream components of the complement pathway are most likely also involved in tauopathy. C5a receptors (e.g. C5aR) were shown to be closely associated with NFTs in human brains [93] and C5aR antagonists decrease tau pathology in 3xTG-AD [90]. Proteins of the MAC are also located on neurons with NFTs [146, 206, 287, 315, 324] and an increase in MAC formation was shown to lead to increased tau pathology and neuron loss [45]. Curiously, however, knocking out C1q - the initiating factor of the classical complement pathway – had no effect on neuroinflammation and tau pathology in the 3xTG-AD mouse model [91]. Collectively, these results show that the multiple parts of the complement pathway regulate tau accumulation and its downstream consequences.

### The role of microglial secreted factors in tau pathology

The mechanism of inflammation-induced tau pathology seems to be at least partly mediated through the direct effect of pro-inflammatory cytokines. The best characterized cytokine involved in this regard is IL1B, which is cleaved into its active form by caspase 1 – downstream of NLRP3 inflammasome activation [276]. Indeed, the inflammasome is robustly upregulated in response to aggregated tau [284]. IL1B increased the accumulation of hyperphosphorylated tau and was associated with reductions in synaptic marker synaptophysin in vitro [169, 185]. This effect was replicated in vivo and a number of studies have now shown using a variety of genetic and pharmacological approaches that this effect was mediated via the inflammasome and ultimately leads to hyperphosphorylation of tau by the kinases cdk5/p25, GSK-3β and p38-MAPK [29, 57, 107, 164, 197, 199, 273]. The cytokine IL-18 is also a product of the NLRP3 inflammasome and was shown to induce kinases that led to tau hyperphosphorylation [230]. The strongest evidence for inflammation-induced initiation of tau pathology currently exists for TNFa. This cytokine is almost exclusively expressed in microglia and can cause formation of tau aggregates in neuronal neurites in vitro via the formation of reactive oxygen species [116]. Furthermore, overexpression of TNFa in 3xTG-AD mice led to increased tau pathology [150]. Knockout of TNF-R2 or both TNF-R1 and TNF-R2 in the same mouse model led to increased plaque and tau pathology [214, 215]. It is therefore possible that both TNFa receptors have complex and opposing effects on the development of tau pathology, but more studies in mouse models of pure tauopathies are needed. As mentioned previously, the cytokine IL6 is also consistently upregulated in tauopathy mouse models. IL6 leads to phosphorylation of tau at AD-associated residues via deregulation of the cdk5/p35 pathway [245]. In addition to the effects of cytokines on kinases and phosphates, it was recently shown that metalloproteinase MMP-9 causes tau aggregation via deacetylase HDAC6 [299]. Furthermore, the leukotrine 5-Lipoxygenase is upregulated in tauopathies, worsens tau pathology, neuroinflammation, and increases synapse loss [58, 108–111, 184, 300, 301]. More studies are needed to identify if and how microglia can initiate tau aggregation, rather than mere aggravation of existing tau pathology.

### The role of microglia in synaptic spreading of tau

Microglia can phagocytose extracellular tau, and aggregated or hyperphosphorylated tau is observed in microglia of mice and humans with tau pathology [37, 38, 44, 73, 99, 192, 196, 216, 229, 238]. Furthermore, microglia can phagocytose synapses or entire neurons that contain aggregated tau [44, 75]. Microglia, however, may also play a critical role in spreading of tau protein [12]. When mice were injected with an adeno-associated virus (AAV) that led to overexpression of human mutated tau in the entorhinal cortex, spreading of human tau from the entorhinal cortex to the dentate gyrus was observed at 1 month post injection. Since neurons in the entorhinal cortex connect to neurons in the dentate gyrus via the perforant pathway, this spreading was likely mediated through synaptic connections. However, depletion of microglia led to a reduction of human tau detected in the dentate gyrus. Knock out of TREM2 adapter protein DAP12 in a similar model also led to inhibition of synaptic tau spreading [14]. Therefore, it will be important to characterize microglial pathways that are involved in opsonization, degradation, and secretion of pathological tau. An interesting recent in vitro study examined the ability of primary microglia derived from various human tauopathy cases or the rTg4510 mouse model to degrade pathological tau [135]. The authors cultured the microglia for multiple days and then applied to conditioned medium a sensitive Förster resonance energy transfer biosensor assay to measure tau seeding activity. Indeed, microglia from human tauopathy cases as well as the rTg4510 mouse secreted seed-competent tau. Microglia also phagocytosed seed-competent tau, however, rather than fully degrading it, they secreted tau back into the extracellular space. Although a portion of tau spreading might be mediated via neuron-to-neuron transfer or via glial cells such as astrocytes [200, 218, 323], available evidence suggests that microglia might play an important role in tau spreading as well.

### Effects of AD risk genes on microglia and tau pathology

Many LOAD risk genes are predominantly expressed in the innate immune system and enriched in microglia [124]. The research on the links between tau pathology and AD risk genes is still at an early stage, with new associations such as BIN1 reported very recently [96]. Studies that have studied the risk factors in the context of neuroinflammation and tau pathology have so far focused on the strongest risk factors: *APOE (apolipoprotein E) ε4* and *TREM2*. *APOEε4* is a common variant of the *APOE* gene and the strongest risk factor for LOAD. *TREM2* risk mutations are substantially less common than the *APOEε4* allele, but their risk effect for LOAD is almost of the same magnitude [276]. Interestingly, two recent studies independently identified a unique *TREM2*-dependent transcriptional network in disease-associated microglia (DAM) that is associated with a wide range of disease and neurodegenerative conditions [161, 172]. Indeed, similar transcriptional networks were described in mouse models of tauopathy [156, 190, 202, 309]. The DAM identity is distinct from the classically described pro-inflammatory microglial phenotype that can be induced by stimuli such as LPS or interferon gamma. Like classic pro-inflammatory microglia, DAM upregulate pro-inflammatory genes (e.g. *IL1B*, *CCL2*) and downregulate homeostatic genes (e.g. *P2ry12*, *Tmem119*). However, in contrast to the LPS-induced microglia, DAM upregulate other genes like *APOE* and *TREM2*. In addition to being part of the DAM genetic network, *TREM2* and *APOE* have also been shown to physically interact with each other and this pathway was important for the phagocytosis of Aβ [276]. Interestingly, APOE was also shown to directly bind to C1q, thereby acting as an immune checkpoint inhibitor of inflammation in response to amyloid plaques [321]. However, the effects of both genes on progression of plaque pathology are complex and dependent on disease stage [276]. The research on the effects of *TREM2* and *APOE* on tau pathology is at an early stage, but the findings so far will be discussed below.

TREM2 is a transmembrane receptor of the immunoglobulin-superfamily that in the brain is predominantly expressed on microglia. Activation of TREM2 leads to interaction with its adaptor protein DAP12 (also known as TYROPB). The ITAM domain of DAP12 recruits SYK, which activates signaling cascades that are involved in metabolism, survival proliferation, and phagocytosis [276]. TREM2, but not DAP12, is progressively upregulated in PS19 mice [153]. Knocking down TREM2 using a lentivirus led to increased levels of pro-inflammatory cytokines, kinases, hyperphosphorylated tau, increased neurodegeneration, and behavioral deficits [153]. Overexpression of murine TREM2 instead of a knockdown led to exactly the opposite phenotype and additional upregulation of homeostatic genes in microglia [154]. Accordingly, knockdown and overexpression of TREM2 in a neuron-microglia co-culture showed that TREM2 prevents the effects of microglial activation and pro-inflammatory signaling on tau phosphorylation [155]. *TREM2* gene knock-out in the mild hTau model that expresses all six human isoforms led to exacerbation of tau pathology [23]. However, knockout of *TREM2* in the more aggressive PS19 mouse model at later stages showed a marked reduction in neurodegeneration and DAM-associated genes [183]. Surprisingly, a recent study using the same conditions showed that TREM2 haploinsufficiency led to more severe tau-induced neurodegeneration compared to the full knockout [261]. Knockout of TREM2 adaptor protein DAP12 in PS19 mice at early disease stages led to increased hyperphosphorylated tau [14], which was also associated with alterations in electrophysiological readouts and cognitive deficits. The data available on TREM2 and downstream effectors (e.g. DAP12 and SYK) thus are contradictory and more studies in different tauopathy models and varying stages of tau-induced neurodegeneration are warranted.

APOE is a lipid carrier that is predominantly expressed in astrocytes and to a lesser degree in microglia. The human brain contains three different alleles: ε2, ε3 and ε4. One copy of ε4 increases AD risk by about 3 times, whereas ε4/ε4 increases risk 12 times [276]. Surprisingly, however, APOEε4-negative prodromal AD patients had greater tau pathology load, cortical atrophy and faster cognitive decline compared to APOEε4 carriers [203, 204]. In AD, APOEε4 only associates with tau pathology in the presence of amyloid pathology [86]. However, in frontotemporal dementia with MAPT mutations that lead to familial tauopathy, APOEε4 lowers the age of onset independent of amyloid plaques [168]. In contrast, another study found that APOEε2 was associated with increased tau pathology burden in PSP [327]. So far, only two studies have experimentally examined the role of different APOE alleles on tau-induced neuroinflammation and neurodegeneration in tau transgenic animals. When PS19 mice were crossed with knock-in mice for the different APOE alleles, the APOEε4 group had the most widespread phospho-tau staining in the hippocampus despite similar levels of insoluble tau. The staining was characterized by a dotted and grainy appearance. This staining pattern was most strongly associated with lower hippocampal volume and was completely absent in the APOE knockout mice. Notably, the APOEε4 group had no dense tangle-like neurons in the phospho-tau staining, but no staining for NFTs was performed in this study. The APOEε4 group also had more severe microgliosis, astrocyte activation and neurodegeneration compared to the APOEε2 and APOEε3 groups [277]. Furthermore, in the same study, APOE knockout mice were less affected on all these measures compared to all the other APOE groups. Intriguingly, a recent study showed dramatically different results when inducing tau pathology using AAVs in knock-in mice for the different APOE alleles [327]. The APOEε2 group had substantially increased tau pathology and showed increased astrocyte reactivity. However, there was no microgliosis or neurodegeneration in any of the APOE groups compared to the control group that just overexpressed GFP. The use of different mouse models potentially representing different stages of tau pathology could explain the apparent discrepancy between these studies. More work, however, needs to be done to determine how different APOE alleles affect tau-induced neuroinflammation and neurodegeneration. For example, microglia expressing APOEε4 display increased phagocytosis of apoptotic neurons [219]. Since APOE is expressed in both astrocytes and microglia, cell-type specific knock-in or knockout models would contribute greatly towards determining the role of different cell types in tauopathy. It would also be particularly informative to further investigate different APOE alleles in various primary tauopathies and tauopathy mouse models at different disease stages. Finally, it is important to keep in mind that APOE has prominent non-immune system related functions and the different APOE alleles therefore likely also influence tau-mediated neurodegeneration via other pathways [20].

## Intersection of tau pathology and microglia at the synapse

### Effects of microglia on tau-induced synaptic dysfunction

Intracellular tau pathology can damage the synapses from within via a multitude of pathways [148]. Aggravation of intracellular tau pathology by microglia can therefore indirectly lead to more tau-induced synapse loss. Microglia, however, can also play a direct role in neurodegeneration-induced synaptic dysfunction (Fig. 2b). One particularly compelling example is reactivation of complement-mediated synaptic pruning, which was first described in neurodevelopment [286]. This pathway starts with synaptic tagging of C1q and downstream synaptic deposition of C3, which leads to opsonization of the synapse via the C3R on microglia [262]. Reactivation of this pathway has been previously demonstrated in multiple mouse models of neurodegenerative disease, including glaucoma [286], FTD [191], and AD [134]. There is also a dramatic upregulation of C1q in normal aging (~ 300-fold in certain brain regions) and age-related cognitive decline was prevented in C1q and C3 KO mice [275, 285]. Additionally, C1q is robustly upregulated in tauopathy patients as it was shown to colocalize with neuronal and astrocytic tau pathology in PiD [279]. Furthermore, C1q is detected alongside hyperphosphorylated tau in AD-derived synaptosomes [75] and decorates both the Aβ plaques and NFT-bearing neurons in AD brain sections [2, 40, 206, 267, 272]. Indeed, complement-mediated pruning of excitatory synapses is strongly re-activated in the PS19 mouse model of tauopathy and this was reversed after intracerebral injection of an anti-C1q antibody [75].

It is unclear how tau pathology leads to C1q-mediated tagging of synapses but a possible pathway could include local apoptotic mechanisms, leading to the exposure of phosphatidylserines on the synapse to which C1q can bind [44, 123]. Furthermore, activation of the metabotropic glutamate receptor 1 was shown to lead to local C1q mRNA synthesis at the synapse in a mouse model of AD. This led to phagocytosis of the synapse by microglia [33]. Additionally, sialic acids in the cell membrane prevent C1q binding and microglia phagocytosis through C3R [189]. It is therefore possible that intracellular tau pathology decreases sialic acid coating on the extracellular side of the synaptic cell membrane. It has been shown recently that TREM2 adaptor protein DAP12 plays an important role in tau-induced induction of C1q [14]. Although the same study could not find similar effects by knocking out TREM2, it would be interesting to study if TREM2 itself could induce synapse opsonization by microglia as has been observed in neurodevelopment [88]. Finally, fibrinogen leakage from blood vessels can also directly lead to microglial phagocytosis of spines via CR3 in mouse models of AD [209]. Tau-induced vascular or BBB damage may therefore lead to increased microglial synapse phagocytosis. More studies, however, are needed to uncover and understand the mechanistic link(s) between tau pathology, C1q-mediated tagging of synapses and microglial phagocytosis of synaptic compartments.

Tau pathology-induced alterations in microglial secreted factors may also adversely affect synaptic function. Microglia in the adult brain are important for learning-induced synapse formation via secretion of neurotrophic factor BDNF [235]. Microglia are known to downregulate many homeostatic genes in response to neurodegeneration, and it is possible that neurotrophic support from microglia to synapses is disrupted in tauopathy [129]. Similarly, tau pathology also induces a pro-inflammatory phenotype in microglia, leading to chronic elevation of pro-inflammatory cytokines. Describing the individual synaptic effects of these cytokines is beyond the scope of this review (see [225]). However, IL1B, IL6 and TNFa have, for example, been shown to modulate various synaptic deficits in mouse models of AD, viral infection, addiction, Creutzfeldt Jakob disease, obesity, and aging [28, 64, 81, 103, 181, 266, 311]. Factors secreted from microglia may also have an indirect effect on synapses. For example, activated microglia secrete extracellular vesicles with miRNAs that downregulate synaptic proteins and ultimately lead to loss of excitatory synapses [243]. Chronically increased levels of pro-inflammatory cytokines and dysregulation of other secreted factors from microglia throughout the decades of developing tau pathology may therefore adversely affect synaptic function in tauopathy patients. The exact contributions of these pathways to tau pathology are still unknown.

### The role of astrocytes in tau-induced synaptic dysfunction

Microglia also have bidirectional signaling cascades with astrocytes. Astrocytes are a highly heterogenous population that make up approximately 20% of brains cells and are derived from the same progenitors as neurons [6]. Astrocytes have a wide range of functions, including providing nutrient support to neurons, forming part of the BBB, and modulating the flow of CSF in the brain as part of the glymphatic system [6, 247]. Astrocytes have highly ramified processes and it is estimated that a single cortical astrocyte can contact up to 100,000 synapses in mice and up to 2,000,000 synapses in humans [5]. Indeed, astrocytes play a critical role in neuronal connections by regulating glutamate homeostasis, secreting gliotransmitters (e.g. ATP), secreting factors that promote assembly and plasticity of synapses (e.g. thrombospondins), and synaptic phagocytosis (e.g. via MERTK and MEGF10) [6]. Under a variety of disease and neurodegenerative conditions, microglial cytokines (IL1a, TNFa and C1q) can induce a unique transcriptional profile in astrocytes that is characterized by dramatic upregulation of complement protein C3. This was associated with a neurotoxic phenotype termed “A1 astrocytes”, characterized by secretion of neurotoxic factors, loss of neurotrophic functions, and impairments in several homeostatic synaptic functions [188]. A1 astrocytes can be induced in normal aging mice and are associated with more severe neurodegeneration in a mouse model of tauopathy [36, 61, 277]. Interestingly, microglia also secrete factors (e.g. TGFα, VEGF-B) that limit the pathogenic activities of astrocytes [253]. Furthermore, C3 upregulation in astrocytes is not only the result of microglial inflammation, but the downstream cleavage product C3a can in turn dramatically increase the synaptic toxicity of microglia in mouse models of amyloidosis and tauopathy by binding to microglial C3aRs [186, 187, 190]. Cross-signaling between microglia and astrocytes therefore plays a key role in modulating synaptic dysfunction and neurodegeneration (Fig. 2b).

Tau pathology can lead to synapse loss via a decrease in neurotrophic thrombospondin signaling by astrocytes [278]. In addition, impaired gliotransmitter release from astrocytes was also shown to mediate tau-induced synaptic dysfunction [239]. It is expected that neurofibrillary pathology-induced loss of astrocytic glutamate homeostasis causes neuronal network dysfunction and potential excitotoxicity. However, an interesting study shows that healthy subjects with NFTs had more activated astrocytes with increased glutamate transporter 1 expression compared to AD cases with dementia [167]. This raises the possibility that at least some astrocytic phenotypes observed in tauopathies may be beneficial rather than damaging. Astrocytes can also prune synapses in the healthy brain [59, 60] or under disease conditions, such as ischemia [217] and sleep deprivation [19]. Furthermore, astrocytes were shown to phagocytose apoptotic cells via the C1q-MEGF10 pathway [143]. This raises the possibility that not only microglia, but also astrocytes can use the classical complement pathway to phagocytose synapses on living neurons. Astrocytes were also shown to clear dystrophic neurites in a mouse model of AD [114]. Since dystrophic neurites in AD patients often contain aggregated tau, it is possible that astrocytes phagocytose pathological tau species. Furthermore, the close proximity of astrocytes to the pre- and postsynaptic compartments also raises the possibility that astrocytes can pick up secreted extracellular tau or digest damaged synapses with hyperphosphorylated tau [73]. Intriguingly, recent studies show that astrocytes may also be involved in tau spreading along neuronal connections or from astrocyte-to-astrocyte [200, 220]. Finally, a transgenic mouse model of astrocytic tau pathology displayed reduced expression and function of glutamate transporter-1, and motor impairments already before disease stages with overt hyperphosphorylated tau accumulation [67]. This indicates that astrocytic tau pathology may lead to alterations in synaptic glutamate homeostasis, neuronal network dysfunction, and associated functional impairments .

## The role of microglia in therapeutic approaches targeting the immune system in tauopathies

Given the increasing recognition of microglia as central players in the pathogenesis of tauopathies, it is perhaps not surprising that there is increasing interest in targeting inflammatory pathways for these diseases (Table 1). Anti-inflammatory compounds such as FK506 and minocycline were shown to reduce tau pathology and downstream neurodegeneration, but their mechanism of action in relation to tau pathology is unclear [104, 226, 322]. Depletion of immune cells such as microglia or T-cells may also be efficacious when initiated at early stages of tau pathology [12, 175]. However, it is important to keep in mind that the immune system in the periphery and the brain plays an important physiological role. Non-specific suppression of the immune system could leave the patient vulnerable to increased risk of infections and accumulation of cellular debris in the context of neurodegeneration. Targeted pharmacological removal of dysfunctional cells from the brain may in itself be an efficacious therapeutic approach for tauopathies [47]. Furthermore, restoring homeostatic microglial phagocytosis with a CD22-blocking antibody rescued cognitive deficits in aged mice, indicating that normal microglial function is critical for maintaining a normal cognitive state [240]. There is therefore increasing focus on targeting specific inflammatory targets such as complement [75, 190], leukotrienes [108, 110], or the NLRP3 inflammasome [284]. Antibodies against C1q can directly reduce tau pathology-induced synapse phagocytosis by microglia [75]. It remains to be determined to what extent microglial synapse phagocytosis plays a central role in the pathogenesis of tauopathies, and if there are other mechanisms through which microglia affect tau-induced synaptic dysfunction that could be targeted with therapeutic agents.

Immunotherapy directly targeting tau protein has also emerged as a promising approach in the treatment of tauopathies and microglia have been increasingly recognized as an important player in the mechanism of action of immunotherapeutic approaches [228]. Active tau immunotherapy has been pursued in multiple varieties: immunization with full-length tau, peptides corresponding to domains on tau, or peptides that correspond to tau phosphorylated at specific residues. Most of these approaches led to reductions in measures of tau pathology, such as sarkosyl insoluble tau and NFTs (Table 2). As tau vaccines trigger an active immune response, patient safety must be carefully monitored. Although in several pre-clinical tau vaccination studies some adverse effects ranging from paralysis caused by severe neuroinflammation to microgliosis have been described [24, 35, 252, 254, 296], these studies used strong T helper 1 inducing adjuvants, which are not used in humans because of safety concerns [63]. Studies that used milder adjuvants have reported similar efficacy, but either no change or reductions in microgliosis [[9, 30, 42, 152, 246, 268] (summarized in Table 2). Passive immunotherapy with monoclonal antibodies offers the advantage of tight control over antibody binding characteristics and titers in the blood. Antibodies binding to specific domains on tau, specific tau phosphorylation sites, pathological tau conformations, and tau oligomers have been developed. Various tau antibodies can decrease sarkosyl insoluble tau, NFTs, and tau spreading (Table 3). Except for one study (see [69]), no differences in gliosis or pro-inflammatory cytokines have been described in passive immunotherapy studies that tested for neuroinflammation [[41, 50, 51, 53, 54, 66, 258] (summarized in Table 3). However, it should be noted that the majority of studies did not report the effects of passive immunotherapy on microglia or other markers indicative of neuroinflammation.

**Table 2:** Preclinical studies using active immunotherapy *in vivo*

Publication	Peptide/adjuvant	Animal model/ immunization start	Immunization schedule	Results
Rosenmann (2006) [252]	Tau1-441 + CFA + PT	C57BL/6 (+/- MOG)	Vaccine, PT 2D later, Tau-CFA 1W later	Tau pathology in neurons and glia, severe *neuroinflammation*, axonal degeneration, tail and hind leg paralysis, behavioral impairments
Boimel (2010) [35]	Tau195-213[P202/205], Tau207-220[P212/214], Tau224-238[P231] peptide mix + MBT + PT	Tau-K257T/P301S (+/- MOG) 4M	Vaccine, PT 2D later, peptides 1W later	↓ NFTs, *microgliosis*, NC *astrogliosis*, changes in lysosomal proteases
Boutajangout (2010) [42]	Tau379–408[pS396/S404] + aluminium phosphate	hTau (6 isoforms) crossed with PS1 M146L 3-4M	See Boutajangout (2010)	↓ P-tau, NC *micro- and astrogliosis*, improved cognition
Bi (2011) [30]	Tau395–406[pS396/S404] + CFA + KLH or IFA	pR5 (4R2N/P301L) 4M, 8M or 18M	0W, 2W and 4W	↓ P-tau, ↓ NFTs, ↑*astrogliosis* in aged group
Rozenstein-Tsalkovich (2013) [254]	See Boimel (2010)	Tau-K257T/P301S 6M or 12M	Vaccine, booster 2W later, peptide mix every month (7x in 12M mice, 4x in aged mice)	Severe *neuroinflammation*, neurological deficits
Theunis (2013) [296]	Tau393-408[pS396/pS404] liposomes	Tau.P301L (2N4R/P301L) 6M	0W, 2W, 4W. Then once after 3M or 2-monthly intervals	↓ P-tau, ↓insoluble tau, NC *micro- and astrogliosis,* improved clinical parameters
Ando (2014) [9]	PHF + aluminium phosphate	THY-Tau22 (4R1N/G272V & P301S) 12M	0W, 2W, 6W, 10W	↓NFTs, ↓insoluble tau, NC *micro- and astrogliosis*
Selenica (2014) [268]	2N4R or 2N4R/P301L + Quil-A	Tg4510 (4R0N/P301L) 5M	0W, 2W, 4W, then 10W rest and followed by 3x 3-weekly boosters	↓ P-tau, ↓*micro- and astrogliosis*
Rajamohamedsait (2017) [246]	See Boutajangout (2010)	3xTg (4R02/P301L, PS1 M146V, APPSWE) 3M	See Boutajangout (2010)	↓ P-tau, ↓ MC1, ↓ insoluble tau, ↓*microgliosis*, NC *astrogliosis*, ↓plaque burden
Benhamron (2018) [24]	See Boimel (2010)	APPSwe/PSEN1dE9-tg 14M	See (Boimel, 2010)	↓P-tau, ↓ Aβ burden, ↑*microgliosis*, NC *astrogliosis*, improved cognition
Ji (2018) [152]	Tau294-305 VLP	PS19 (4R1N/P301S) 3M	4x at 2-weekly or 3-weekly intervals	↓ P-tau, ↓ insoluble tau, ↓*microgliosis*, ↓*astrogliosis*, ↓synapse loss, improved cognition

**Table 3:** Preclinical studies using passive immunotherapy *in vivo*

Publication	Antibody/epitope	Animal model/ immunization start	Duration/interval/dose/ROI	Results
Chai (2011) [54]	PHF1 (p396/p404) & MC1 (conformational)	JPNL3 (4R0N/P301L) 2M & PS19 (4R1N/P301S) 2M	**JPNL3** 2M, 3x/week (15mg/kg i.p.) then 2M, 2x/week (10mg/kg i.p.) & **PS19** 2M, 2x/week (15mg/kg i.p.)	↓ P-tau, NC *micro- and astrogliosis*, ↓ weight loss & motor impairment
Boutajangout (2011) [41]	PHF1	JPNL3 2-3M	3M, 1x/week (250ug/mouse i.p.)	↓ P-tau, ↓insoluble tau, NC *astro- and microgliosis*, improvement traverse beam task, NC rotarod
D'Abramo (2013) [66]	PHF1, MC1 & DA31 (aa150-190)	JPNL3 3M, 6M & 7M	4M, 1x/week (250ug/mouse/i.p.) & survival analysis, 1x/week (250ug/mouse i.p.)	Only MC1 effective. ↓ P-tau, ↓insoluble tau, NC survival, NC *micro- and astrogliosis*
Castillo-Carranza (2014) [53]	TOMA (conformational)	JPNL3 8M	Single injection (30ug/mouse i.v. & 1ug/mouse i.c.v)	↓Tau oligomers, NC *microgliosis* and cytokines, improved cognition & motor deficits
Castillo-Carranza (2014) [50]	TOMA	hTau (6 isoforms) 3M (injected with tau oligomers)	Single injection & 6M complex schedule (60ug/mouse i.v. )	↓tau oligomers, NC inflammation, improved cognition
Castillo-Carranza (2015) [51]	TOMA	Tg2576 (APPSWE) 14M	Single injection (30ug/mouse i.v.)	↓tau oligomers, ↓Aβ oligomers, ↑ plaques, NC *microgliosis* and cytokines, NC synapse loss, improved cognition
Sankaranarayanan (2015) [258]	PHF6 (p231) & PHF13 (p396)	rTg4510 (4R0N/P301L) 3M & PS19 (injected with PFF)	**rTg4510** 3M, 1x/week (25mg/kg i.p.) & **PS19** 4W, 1x/week (30mg/kg i.p.)	**rTg4510:** ↓Soluble P-tau, NC insoluble tau, NC *inflammation*, NC or improved cognition. **PS19-PFF:** ↓Tau spreading, improved cognition
Dai (2017) [69]	43D (aa6-18) & 77E9 (aa184-195)	3xTg (4R02/P301L) 12M	2W & 6W, 1x/week (15ug/mouse i.v.)	↓P-tau, improved cognition, ↑*activated microglia morphology*, ↑complement (C1q & C9), ↓plaques

Most research has focused on the development of antibodies that neutralize extracellular tau and inhibit synaptic spreading of pathological tau [227, 320]. Tau antibodies could neutralize extracellular tau oligomers before they have the chance to damage glial cells and the vasculature. As mentioned previously, both microglia and astrocytes are involved in propagation of tau pathology [12, 200]. Antibody-mediated neutralization of tau seeds before they reach these types of cells, may therefore diminish downstream exosomal tau spreading. Additionally, extracellular tau aggregates may lead to reactive gliosis, which can potentially be inhibited by anti-tau antibodies. Extracellular tau aggregates neutralized by tau antibodies need to be removed from the brain. Clearance of extracellular tau or other macromolecules is mediated by the glymphatic system and impairment of this system worsens tau pathology [[141, 236]. In addition to global clearance, tau-antibody complexes in immunized mice can also be cleared locally by means of opsonization via microglial Fc receptors and degradation in the lysosomes [8, 10, 74, 99, 179, 319]. Effectorless antibodies incapable of actively engaging microglia retained their therapeutic effect [179], indicating that tau antibody complexes can also be cleared via additional pathways in immunized mice. Indeed, peripheral or AAV-mediated delivery of tau scFv (without an Fc domain) seems to be an effective therapeutic approach in tauopathy mice [145, 224, 283, 306]. More research is therefore needed to describe the impact of actively engaging microglia with tau immunotherapeutic approaches.

## Concluding remarks & future directions

Microglia are fascinating cells and the large number of excellent recent studies demonstrates increasing recognition of microglia as critical players in the pathobiology of tau protein. It is currently not fully resolved if inflammation is a cause, contributor or consequence of tau pathology. Pro-inflammatory mediators secreted from microglia (e.g. cytokines, complement) can initiate tau pathology and play a critical role in tau-induced neurodegeneration. The strongest evidence for inflammation-induced initiation of tau pathology currently exists for TNFa, as this cytokine was shown to lead to formation of tau aggregates in neurites [116]. More preclinical work, however, is needed to fully characterize the immune pathways involved in tau pathology and efforts should be made to validate them in both AD and primary tauopathy patients. Many studies report the effects of neuroinflammatory processes on tau phosphorylation only. Future studies should also focus on the effects of neuroinflammation on oligomerization or accumulation of insoluble tau aggregates. Furthermore, risk genes for AD or other tauopathies will have to be investigated in multiple mouse models of tauopathy, without plaque pathology as a confounding factor. Since the immune response to tau pathology changes as the disease progresses, future studies should also examine the evolution of neuroinflammatory pathways at multiple stages of the disease. Furthermore, more studies are needed on what events cause the initial neuroinflammation in response to tau pathology and via what pathways.

Microglia are also directly involved in tauopathies as they have been shown to pathologically phagocytose synapses of neurons with tau pathology. Currently, the pathways underlying microglia-mediated synapse loss are not fully characterized and a multitude of potential pathways have been identified in neurodevelopment. Complement-mediated synapse loss via microglial synapse phagocytosis under neurodegenerative conditions is now described in multiple disease models. However, it is not known what causes the binding of C1q to synapses and if this happens indiscriminately or only targets vulnerable synapses. In neurodevelopment, there are signals (e.g. CD47) that protect synapses from microglial pruning. We need to understand better the function of these signals in the normal brain and determine if they are downregulated in response to neurofibrillary pathology. Additionally, since astrocytes play a critical role in both synaptic function and neuroinflammation, more studies are needed on bidirectional microglia-astrocyte signaling in tauopathies.

As principal macrophages of the brain, microglia phagocytose tau and may play a role in spreading tau pathology throughout the brain. Determining the exact contribution of microglia to the disease pathogenesis remains an important topic for future investigations. More studies are needed on the mechanisms of tau internalization in microglia and if this is associated with activation of pro-inflammatory pathways. Furthermore, better identification of intracellular pathways that lead to degradation of tau aggregates in microglia is required. This could help explain why microglia in tauopathies are mostly senescent, inefficient in clearing extracellular tau, and potentially become involved in spreading of tau pathology. The loss of the ability of microglia to keep the extracellular space clean of aggregated proteins may play a critical role in the propagation of protein pathology in the brain.

Finally, the role of microglia in the mechanism of action of tau immunotherapy needs to be explored further. Important topics include a better understanding of the fate of tau-antibody complexes in the extracellular space and the associated roles of various antibody isotypes; a better understanding of microglia-mediated clearance mechanisms and other potential clearance pathways like the glymphatic system; and a better characterization of the downstream consequences of Fc-mediated tau antibody internalization. Pre-clinical studies should also report more on the immunological consequences, beyond the mere presence of microgliosis or astrogliosis. Examples would include measuring secreted immune molecules (pro-inflammatory cytokines, complement) and identifying effects on transcriptional phenotypes in glia, such as the recently identified disease-associated microglia and neurotoxic A1 astrocytes [161, 188]. The field of neuroimmunology is advancing rapidly with insights that revolutionize our thinking about the microglia in the brain. Applying these insights to the study of tau pathology will pave the way towards better understanding and treatment of tauopathies.

## Data Availability

Not applicable.

## Abbreviations

AAV: Adeno-associated virus
AD: Alzheimer’s disease
AGD: Argyrophilic grain disease
APOE: Apolipoprotein E
APP: Amyloid precursor protein
Aβ: Amyloid beta
BDNF: Brain-derived neurotrophic factor
C: Complement
C3R: Complement receptor 3
CBD: Corticobasal degeneration
CD47: Cluster of differentiation 47
CFA: Complete Freund adjuvant
CSF: Cerebrospinal fluid
CTE: Chronic traumatic encephalopathy
Cx3cl1: Chemokine (C-X3-C motif) ligand 1
Cx3cr1: CX3C chemokine receptor 1
DAM: Disease-associated microglia
Fc: Fragment crystallizable
GWAS: Genome-wide association studies
I.c.v.: Intracerebroventricular
i.p.: Intraperitoneal
i.v.: Intravenous
IFN: Interferon
IL: Interleukin
LOAD: Late onset Alzheimer’s disease
LPS: Lipopolysaccharide
LTP: Long term potentiation
MAPT: Microtubule-associated protein tau
MBT: Mycobacteriumtuberculosis H37Ra
MOG: Myelin oligodendrocyte glycoprotein
MTBR: Microtubule binding repeats
NC: No change
NFT: Neurofibrillary tangle
PFF: Preformed fibrils
PiD: Picks’ disease
PS/PSEN: Presenilin
PSP: Progressive supranuclear palsy
PT: Pertussis toxin
P-tau: Hyperphosphorylated tau
PTM: Post-translational modification
scFv: Single-chain variable fragment
TNF-a: Tumor necrosis factor alpha
TREM2: Triggering receptor expressed on myeloid cells 2
V BBB: Blood brain barrier
VLP: Virus-like particles
